# Development of *Aspergillus oryzae* BCC7051 as a Robust Cell Factory Towards the Transcriptional Regulation of Protease-Encoding Genes for Industrial Applications

**DOI:** 10.3390/jof11010006

**Published:** 2024-12-25

**Authors:** Sarocha Panchanawaporn, Chanikul Chutrakul, Sukanya Jeennor, Jutamas Anantayanon, Kobkul Laoteng

**Affiliations:** Functional Ingredients and Food Innovation Research Group (IFIG), National Center for Genetic Engineering and Biotechnology (BIOTEC), National Science and Technology Development Agency (NSTDA), Thailand Science Park, Phahonyothin Road, Khlong Nueng, Khlong Luang, Pathum Thani 12120, Thailand; sarocha.pan@biotec.or.th (S.P.); sukanya.jee@biotec.or.th (S.J.); jutamas.ana@biotec.or.th (J.A.); kobkul@biotec.or.th (K.L.)

**Keywords:** *Aspergillus oryzae*, PrtR, transcription regulator, protease, esterase, biotechnological potential, fungal biotechnology

## Abstract

Enzyme-mediated protein degradation is a major concern in industrial fungal strain improvement, making low-proteolytic strains preferable for enhanced protein production. Here, we improved food-grade *Aspergillus oryzae* BCC7051 by manipulating the transcriptional regulation of protease-encoding genes. Genome mining of the transcription factor *AoprtR* and computational analysis confirmed its deduced amino acid sequence sharing evolutionary conservation across *Aspergillus* and *Penicillium* spp. The AoPrtR protein, which is classified into the Zn(II)2-Cys6-type transcription factor family, manipulates both intra- and extracellular proteolytic enzymes. Our transcriptional analysis indicated that the regulation of several protease-encoding genes was AoPrtR-dependent, with AoPrtR acting as a potent activator for extracellular acid-protease-encoding genes and a likely repressor for intracellular non-acid-protease-encoding genes. An indirect regulatory mechanism independent of PrtR may enhance proteolysis. Moreover, AoPrtR disruption increased extracellular esterase production by 2.55-fold, emphasizing its role in protein secretion. Our findings highlight the complexity of AoPrtR-mediated regulation by *A. oryzae*. Manipulation of regulatory processes through AoPrtR prevents secreted protein degradation and enhances the quantity of extracellular proteins, suggesting the low-proteolytic variant as a promising platform for the production of these proteins. This modified strain has biotechnological potential for further refinement and sustainable production of bio-based products in the food, feed, and nutraceutical industries.

## 1. Introduction

*Aspergillus oryzae* is widely exploited in traditional Asian food and food-processing industries and is listed as Generally Recognized as Safe (GRAS) [1]. Industrial applications of *A. oryzae* have expanded to several sectors, including the production of organic acids, bioactive molecules, and enzymes. *A*. *oryzae* stands out due to its substrate utilization, stress tolerance, high growth rate, and eukaryotic post-translational modification and secretion capacities, which are advantageous for biometabolite manufacturing. Recently, there has been a growing interest in enhancing the *A*. *oryzae* platform for industrial production using synthetic biology. Several genetic tools, along with omics technology, have supported the holistic development of filamentous fungal metabolic traits for industrial purposes. Strategies to establish a fungal system for industrial-scale production include a robust cell chassis, genetic toolboxes, and precision fermentation.

As *A. oryzae* BCC7051 is a recognized GRAS strain with accessible omics data [2], it was selected based on its superior growth and development across a range of substrates and its ability to produce lipid-rich biomass through submerged fermentation [3]. In addition, this strain has recently been developed into a fungal production system. An antibiotic-free selection system, morphological control, and efficient gene-targeting strategies have been implemented to support fungal chassis development [4,5]. We enhanced the gene targeting efficiency of this strain by disrupting LigD (a homolog of human Lig4), as it has been previously reported for *A. oryzae* [6]. Moreover, biomass production can be improved by modulating ammonium uptake capacity and nitrogen metabolism [7]. Beyond a robust cell chassis, a stable constitutive or inducible expression system is essential not only for protein expression but also for metabolic pathway engineering in this modified strain [8,9,10]. *A. oryzae* BCC7051 has also been employed as a cell factory through genetic manipulation and bioprocessing, demonstrating its considerable potential for producing metabolic products with industrial applications [11,12,13,14,15].

Lipid-degrading enzymes are among the most important biocatalysts that drive demand in many industrial sectors. Yeasts and filamentous fungi are effective production systems for these enzymes, which include several classes of lipases and esterases [16,17] with biotechnological potential [18,19]. Among these microbes, *A. oryzae*, an oleaginous fungus, has demonstrated a diversity of lipid-degrading enzymes through genome mining [20]. This fungus harbors 50 lipase-encoding genes distributed across six lipase gene families, with the carboxylesterase family (EC 3.1.1.1) comprising the majority (52%) of these genes and involved the hydrolysis of ester-containing compounds [21]. The majority of enzymes in this family are secreted, as indicated by putative signal peptide sequences [20]. Consequently, oleaginous *A. oryzae* is emerging as a vital fungal system for the production of industrial lipolytic enzymes.

The industrial application of fungal systems is limited by the enzymatic activity that induces protein degradation [22,23,24]. To address this issue, the reduction in protease production was explored. Genomic analysis of *A. oryzae* RIB40 revealed more than 130 protease-encoding genes, representing approximately 1% of the total genes [25]. Disruption of enzyme-encoding genes has resulted in protease-deficient strains, minimizing protein degradation [26,27,28,29]. Proteolytic degradation is also transcriptionally regulated. The transcriptional regulators PrtT and PrtR, which contain Zn(II)2-Cys6 binuclear DNA-binding sites, were identified [30] and were shown to activate multiple protease-encoding genes. Disrupting *prtT/prtR* in *Penicillium oxalicum* and various *Aspergillus* spp. decreased protease-encoding gene expression, reducing or eliminating protease activity [31,32,33,34]. Moreover, enhanced heterologous protein secretion was observed in *A. niger prtT*-deficient strains [35]. Despite advancements, the improvement of industrial *A. oryzae* strains to reduce protein degradation via protease-encoding gene regulation remains incomplete. A recent study reported the alteration of extracellular peptidase gene transcription and extracellular acid peptidase activity mediated by PrtR in response to the growth environment in *A. oryzae* RIB40 [36].

Therefore, in this study, we aimed to improve food-grade *A. oryzae* BCC7051 by manipulating protease gene transcriptional regulation. We characterized the *prtT* ortholog *AoprtR*, investigated its role in proteolytic enzyme production via gene disruption and overexpression, analyzed the transcriptional levels of selected intra- and extracellular protease-encoding genes in an *AoprtR*-deficient strain, and assessed protease deficiency’s impact on lipolytic enzyme esterase secretion. Our findings demonstrate AoPrtR’s effectiveness in the transcriptional control of protease production. The *AoprtR*-deficient strain exhibited remarkably increased extracellular esterase activity. Our work provides a rational strategy to improve the *A. oryzae* fungal production system using a knowledge-based approach to transcription regulation and cell physiology. Reducing proteolytic enzyme production in *A. oryzae* BCC7051 will facilitate the development of optimized strains and bioprocesses for extracellular protein production in both fundamental and applied research using fungal biotechnology and synthetic biology tools.

## 2. Materials and Methods

### 2.1. Microbial Strains and Cultivation

We used the *pyrG* auxotrophic strain of *A. oryzae* BCC7051 [11] as a basal strain for genetic manipulation. We generated the *pyrG* gene complementation of the auxotrophic strain and the *AoprtR* gene disruption with *pyrG* gene complementation of the auxotrophic strain as the parental (Δ*pyrG::pyrG*) and *AoprtR*-deficient (Δ*pyrG*,Δ*prtR::pyrG*) strains, respectively. Furthermore, we generated an *AoprtR* overexpressing strain, as described in Section 2.3. We maintained these strains on Czapek Dox (CD) medium (BD Difco) and prepared spore inoculums by growing them on steamed polished rice grains at 30 °C for 5–7 days, harvesting the spores using 0.05% (*w/v*) Tween 80 solution. For submerged cultivation, we inoculated the spore suspension at a final concentration of 10^6^ spores/mL into 50 mL semi-synthetic medium, SM (40 g/L (*w/v*) glucose, 0.2 g/L (*w/v*) NH_4_Cl, 5 g/L (*w/v*) yeast extract, 2.4 g/L (*w/v*) KH_2_PO_4_, 0.5 g/L (*w/v*) MgSO_4_∙7H_2_O, 0.1 g/L (*w/v*) CaCl∙2H_2_O, 15 mg/L (*w/v*) FeCl_3_∙7H_2_O, 10 mg/L (*w/v*) MnSO_4_∙H_2_O, and 7.5 mg/L (*w/v*) ZnSO_4_∙7H_2_O) [37] and incubated the culture at 30 °C on a rotary shaker at 200 rpm.

We grew *Escherichia coli* DH5α (Thermo Fisher Scientific, Waltham, MA, USA) in Lysogeny Broth (LB) containing 100 mg/mL ampicillin at 37 °C with shaking at 200 rpm.

We cultured the *Saccharomyces cerevisiae* strain INVSCI (Invitrogen, Waltham, MA, USA) as a host for DNA assembly at 30 °C in a YPD medium under shaking at 200 rpm. We used SD agar supplemented with L-tryptophane, L-histidine-HCl, and L-leucine at concentrations of 0.02, 0.02, and 0.03% (*w/v*), respectively, for yeast transformant selection.

### 2.2. AoprtR Ortholog Identification

Using the *prtR* sequence of *A*. *oryzae* RIB40 (accession number A7BJS7.1) as a query, we conducted a BLASTN search against A. *oryzae* BCC7051 (accession number OOO09832.1) [2] and identified a putative *prtR* regulator-encoding gene. To synthesize prtR cDNA, we extracted total RNA from mycelial cells using a PureLink RNA Mini Kit (Thermo Fisher Scientific, Waltham, MA, USA). We generated first-strand cDNA using qPCR RT Master Mix and eliminated genomic DNA using ReverTra Ace™ and gDNA Remover (Toyobo, Osaka, Japan). We then synthesized the cDNA by RT-PCR using the purified RNA as a template, a specific primer set, and the Super Script III One-Step RT-PCR with Platinum Taq DNA Polymerase (Invitrogen, Waltham, MA, USA). Next, we purified the cDNA for sequence analysis (Macrogen, Seoul, Republic of Korea) and deposited the *AoprtR* sequence in the GenBank database (http://www.ncbi.nlm.nih.gov, accessed on 15 January 2024) under the accession number PP129537. We used the CLUSTAL Omega (1.2.4) program (http://www.clustal.org/omega/, accessed on 12 September 2024) [38], a multiple sequence alignment program for proteins, to determine the identity of prtT/prtR across species and generate a phylogenetic tree.

### 2.3. AoprtR Disruption and Overexpression Plasmid Construction

Figure S1A,C present schemes summarizing disruption and overexpression plasmid construction, respectively. We constructed a disruption plasmid using PCR and DNA assembly in yeast cells [39]. We amplified specific 5′- and 3′-DNA fragments with homologous *AoprtR* sequences using *A. oryzae* BCC7051 genomic DNA as a template, Platinum^TM^ Taq DNA polymerase, High Fidelity (Invitrogen), and overlapping primer sets (Table S1). We assembled homologous DNA fragments and linearized the *AopyrG* marker gene-carrier pAopyrG backbone plasmid in *S*. *cerevisiae* cells. Next, we shuttled the circular plasmid into *E. coli* cells for plasmid propagation and validated the constructed pDAoPrtR plasmid by restriction enzyme analysis and DNA sequencing. We constructed an overexpression plasmid similar to that described above. We cloned the *prtR* cDNA fragment between the 5′- and 3′-DNA fragments with homologous *AopyrG* sequences in the backbone plasmid using a DNA assembly technique. We validated the constructed pOEAoPrtR plasmid as described above.

### 2.4. Fungal Transformation and Genetic Verification

We prepared the DNA fragment of the pDAoPrtR plasmid (2–5 μg) using specific restriction enzyme digestion, purified it, and transformed it into *A. oryzae* protoplast cells using the PEG-mediated transformation method (PMT) [10]. We selected fungal transformants on CD medium by incubating at 30 °C for 5–7 days. We re-isolated the spores to obtain a purified culture. We verified targeted *prtR* gene disruption by PCR using transformant genomic DNA, Phire Plant Direct PCR Master Mix (Thermo Fisher Scientific, Waltham, MA, USA), and specific oligonucleotide primer pairs (Table S2).

### 2.5. Fungal Transformant Growth Characteristics on Solid Agar Plates

We performed a skim milk degradation assay to assess extracellular protease production. We prepared skim milk agar using 10% (*w/v*) skimmed milk powder and autoclaved it at 115 °C for 10 min. We prepared 0.5% peptone and 2% agar solutions separately and autoclaved them at 121 °C for 20 min [40]. Next, we mixed the two solutions and poured them into Petri dishes. The Δ*prtR* transformant and parental spore solutions (10^5^ spores) were inoculated on skim milk agar and incubated at 30 °C for 2–3 days. We detected protease secretion in a clear zone in the medium surrounding the colony. We analyzed the growth diameters and spore production of both strains on PDA plates after incubation at 30 °C for 5 days.

### 2.6. Reverse Transcription Real-Time Quantitative PCR (RT-qPCR) Analysis

In this study, we selected 26 candidate genes encoding proteolytic enzymes (Table 1). Seventeen of these genes, which encode functional proteolytic enzymes active across acid to alkaline pH levels, have been reported to be regulated by PrtT in filamentous fungi [31,36,41,42]. In addition, 9 putative protease-encoding genes from the *A. oryzae* BCC7051 genome were selected. Each gene is a homolog of a known protease-encoding gene, sharing ≥45% amino acid sequence identity. These homologs are listed in Table 1, shown as genes *h1* or -*h2*. We predicted the signal peptides and their corresponding cleavage site locations using the SignalP 6.0 server (https://services.healthtech.dtu.dk/services/SignalP-6.0/, accessed on 7 April 2023) [43]. For differential gene expression analysis, we cultured fungi in SM medium at 30 °C with shaking for 72 h. We extracted total RNA, synthesized cDNA, and subjected the synthesized cDNA to RT-qPCR analysis using a Universal qPCR master mix (Luna^®^, NEB, New England Biolabs (NEB), Ipswich, MA, USA) with specific oligonucleotide primer sets (Table S3). We quantified the relative transcriptional levels by normalizing to 18S rRNA [44].

### 2.7. Cell Growth and Residual Sugar Content

We assessed fungal strain growth profiles and biomass production in the culture broth as follows: we grew mycelial cells on SM medium and harvested them at different culture times by filtration with gentle suction through Miracloth (MerckMillipore, Darmstadt, Germany), followed by drying in a hot air oven at 60 °C until the obtention of constant weight.

We quantified the residual sugar concentration in the culture broths using a high-performance liquid chromatography system (Ultimate 3000, Thermo Fisher Scientific, Waltham, MA, USA) equipped with a refractive index detector. We filtered the culture broths through a 0.2 μm PTFE filter and then diluted them by ten times. Using an Aminex^®^ HPX-87H column (Bio-Rad Laboratories, Hercules, CA, USA), we performed the analysis at 60 °C at a flow rate of 0.6 mL/min using 18 mM sulfuric acid solution as the mobile phase. The pH of the culture broth was measured throughout the experiment.

### 2.8. Analysis of Protease Activity

To measure extracellular proteolytic activity in the culture medium, we harvested triplicate samples of cell-free broth by filtration through Miracloth and centrifugation. We measured the enzymatic activity in the supernatant using the modified Folin–Ciocalteu’s phenol reagent method [45,46,47] with lactate buffer pH 3.5, phosphate buffer pH 7.5, or borate buffer (pH 10.5) as acid, neutral, or alkaline conditions, respectively. We prepared the control (blank) by adding the culture filtrate after trichloroacetic acid. For intracellular proteolytic activity measurement, we smashed the harvested mycelia with liquid nitrogen, extracted them with different buffers for acid, neutral, or alkaline conditions, and performed the assay. Next, we generated a standard curve using 0.05–0.55 µmol of tyrosine. We defined one unit of protease activity as the enzyme required to liberate 1 µmol of tyrosine per minute under the aforementioned conditions.

### 2.9. Esterase Activity Analysis

For the esterase assay, we measured the enzymatic activity as previously described [48] using *p*-nitrophenyl butyrate (*p*-NPB) as a substrate, with modifications. To eliminate the spontaneous hydrolysis effect of *p*-NPB, we used a heat-inactivated enzyme solution as a control. In addition, we prepared and measured standard *p*-nitrophenol (*p*-NP) solutions at various concentrations using the same procedure. We defined one unit of esterase activity as the amount of enzyme required to release 1 µmol of *p*-NP per minute under the assay conditions.

### 2.10. Statistical Analysis

We performed statistical analyses using SPSS software version 11.5 for Windows (SPSS Inc., Chicago, IL, USA). All experiments were conducted using three biological replicates. The data are presented as the mean value with standard error (mean ± SE). We assessed the differences between the means of the two variables using Student’s *t*-test; *p* < 0.05 was considered statistically significant.

## 3. Results and Discussion

### 3.1. A. oryzae BCC7051 prtR Identification and Molecular Characterization

Using *A. oryzae* RIB40 *prtR* as a query, we performed BLASTN analysis and identified a 2137-bp single copy of the *AoprtR* homologous gene from the *A. oryzae* BCC7051 genome data [2] containing four predicted introns. We designated the *AoprtR* cDNA sequence-derived deduced amino acid sequence as AoPrtR. The AoPrtR protein contained 624 amino acid residues with a calculated molecular weight of 70.797 kDa. We classified it as a transcription factor family member with the distinct feature of a Zn(II)2-Cys6 binuclear DNA-binding motif (CNTCRKLKTRCDLDPRGHACRRCLSLRIDC) [34] at amino acid residues 52–81 of the polypeptide. Based on multiple sequence alignment analysis, the deduced amino acid sequence of *AoprtR* shared an identity of 50.1–100% with the PrtT/PrtR sequences of *Aspergillus* spp. and *Penicillium* spp. We observed that the sequence was conserved within these two fungal genera but extensively low in other filamentous fungi, including entomopathogenic fungi. We discovered significant identity among the *A. oryzae* sequences with 99.8% to 100% identity, including RIB40, 3.042, IF04177, and *Aspergillus flavus* (accession numbers: A7BJS7.1, EIT76196.1, CS407838.1, and KAB8242731.1, respectively), as well as *Aspergillus niger* CBS 513.88 (accession number: XP_001402055), two *Aspergillus fumigatus* (accession numbers: CS407841.1 and KMK54744.1), and *Aspergillus awamori* (accession number: GCB23536.1), with 65.2–71.1% identity. In addition, the amino acid sequence displayed 50.1% identity to *Penicillium chrysogenum* and *Penicillium rubens* (accession numbers: CS407843.1 and B6HRH5.1). A phylogenetic tree illustrating the identity of *prtR* or *prtT* across species is presented in Figure S2, which was generated using the CLUSTAL Omega (1.2.4) program. Based on these results, the presence of *prtR/prtT* indicated specific evolutionary gene-conservation characteristics among *Aspergilli* and certain *Penicillium* spp., coinciding with the conclusions of a previous study [49]. In addition, we documented the absence of PrtT/PrtR orthologs in *Aspergillus nidulans*, potentially due to an evolutionary gene loss event through fungal adaptation [33]. *prtT* loss occurred independently during *Aspergillus* speciation, which has been postulated [49].

### 3.2. Effects of AoPrtR on A. oryzae Proteolytic Enzyme Activity

**Figure 1:**
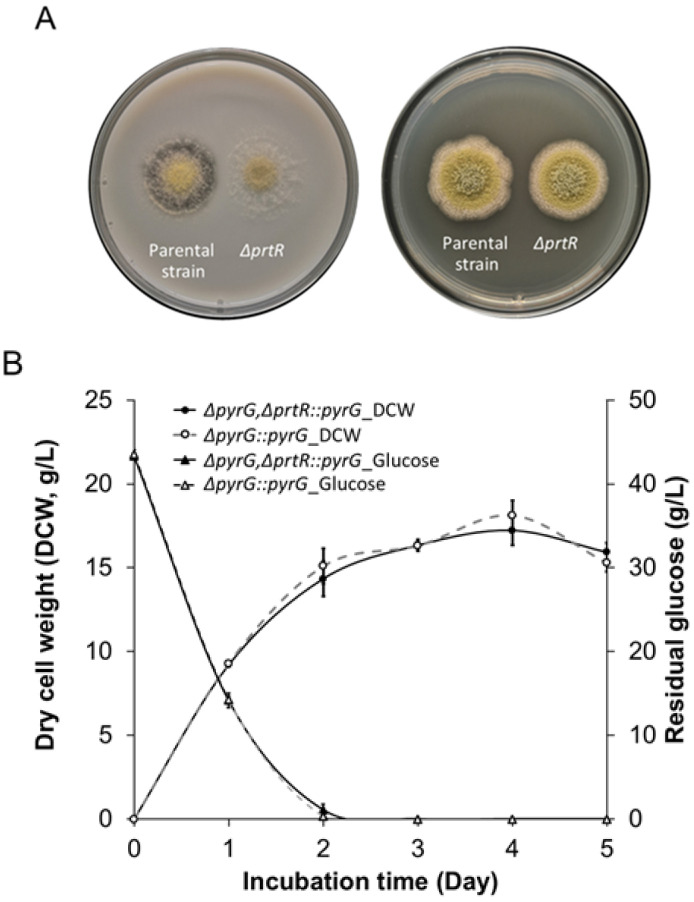
Growth characteristics of the *AoprtR*-deficient *A. oryzae* strain. (**A**) Colonial growth and protease secretion of the *AoprtR*-deficient (Δ*pyrG*,Δ*prtR::pyrG*) and parental (Δ*pyrG::pyrG*) strains grown on skim milk agar (left panel) and PDA (right panel) media. (**B**) Mycelial growth and residual glucose profiles of the *AoprtR*-deficient strain (solid line) and the parental strain (dotted line) in SM broth at 30 °C and 200 rpm. Symbols indicate dry cell weight (circles) and residual glucose concentration (triangles). The data are represented as the mean value with standard error (mean ± SE).

Because of the presence of several proteases in *A. oryzae*, the regulation of these proteolytic enzymes can be a strategic approach to improving strains for homologous and heterologous protein production. To investigate the function of the *A. oryzae* putative AoPrtR regulator, we performed the targeted disruption of *AoprtR* using gene replacement of the *pyrG*-deficient strain. We constructed the pDAoPrtR disruption plasmid and then verified it using restriction enzyme analysis (Figure S1A,B). We subjected the fungal transformants with *pyrG* complementation that could grow on the selective medium to secondary screening using PCR (Figure S3A). Our RT-PCR analysis revealed that the *AoprtR* transcript could not be detected throughout the experiment using the deficient strain (Figure S4), confirming that we obtained an *AoprtR*-deficient strain. We further analyzed a selected true transformant by growing it on a solid medium for assessment of extracellular protease production. The transformant exhibited a protease-deficient phenotype (Figure 1A, left panel), as indicated by the lack of a halo zone when grown on skim milk agar. The colonial growth diameter was consistent with the parental strain when grown either on skim milk agar or on a PDA plate (Figure 1A, left and right panels, respectively). Moreover, the spore production of the deficient strain was comparable to that of the parental strain, i.e., 2.30 ± 0.35 × 10^8^ and 2.41 ± 0.62 × 10^8^ spores/mL, respectively. Cultivation in liquid cultures revealed that the growth profile of this protease-deficient strain was comparable to that of the parental strain, as indicated by biomass titers and residual glucose concentrations (Figure 1B). In addition, pH changes were observed in both strains by decreasing from the initial pH of 5.5 to 3.9 during the log phase and gradually increasing to approximate ≥5.7–6.0 in the stationary phase (Table S4). In conclusion, these results suggested that the alterations in mycelial growth, glucose consumption, and pH values of the deficient culture were comparable with those of the parental strain grown in the liquid culture. These phenotypic characteristics indicate that *AoprtR* disruption does not affect the vegetative growth stage of *A. oryzae*. The *AoprtR* disruption-induced impaired protein degradation phenotype of the transformant revealed the function of AoPrtR as a protease regulator.

**Figure 2:**
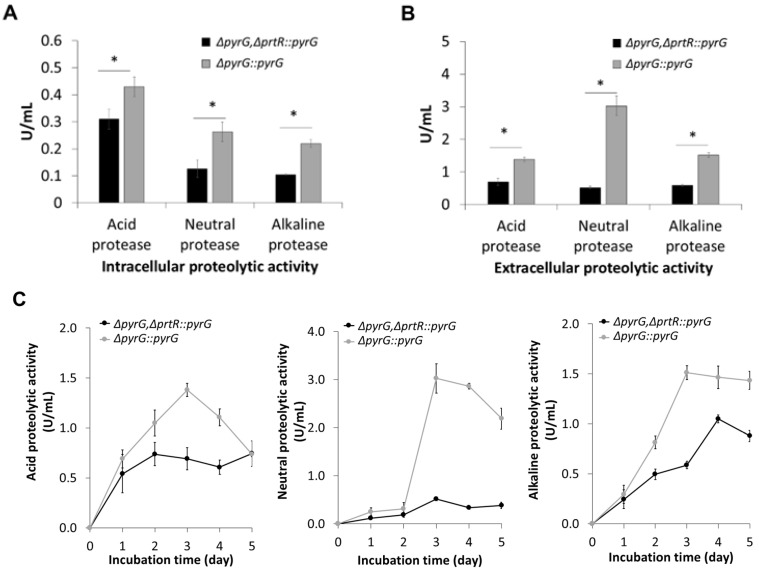
Proteolytic activities of the *AoprtR*-deficient *A. oryzae* strain. (**A**) Intracellular and (**B**) extracellular proteolytic activities of the *AoprtR*-deficient (Δ*pyrG*,Δ*prtR::pyrG* as black bar) and parental (Δ*pyrG::pyrG* as gray bar) strains after 3 days of culture in SM broth at 30 °C and 200 rpm. Asterisks above the bars indicate statistically significant differences in proteolytic activity between strains (*p* < 0.05). (**C**) The time course of extracellular proteolytic activities in the *AoprtR*-deficient (black line) and parental (gray line) strains was measured at different culture times. The left, middle, and right panels represent acidic, neutral, and alkaline proteolytic activities, respectively. The data are represented as the mean value with standard error (mean ± SE).

Next, we analyzed the proteolytic activities of the AoprtR-deficient and parental strains grown in a liquid medium. We observed significantly reduced intra- and extracellular protease activities in the *AoprtR*-deficient strain compared to those of the parental strain, i.e., the control (Figure 2A and Figure 2B, respectively). All protease activities were dominantly present in the extracellular cultures of the deficient strain. Significantly, we observed a 3–6-fold reduction in extracellular neutral and alkaline protease activity in the deficient strain compared to the parental strain (Figure 2B). Notably, the lack of *AoprtR* also influenced the reduction in extracellular acid protease activity in *A. oryzae* BCC7051, as reported in *A. oryzae* RIB40 [26], although to a lesser extent (approximately 50%) (Figure 2B). The specific function of PrtT restricted to protease secretion has been described in certain *Aspergillus* species, including *Aspergillus aculeatus* [32]. In addition, intracellular neutral and alkaline protease activities were reduced by 2–2.2-fold. From these results, *AoprtR* disruption in *A. oryzae* BCC7051 displayed strong attributes not only in extensively reducing extracellular proteolytic enzyme production but also those of the intracellular compartment, particularly neutral and alkaline proteases. These enzymes are responsible for most proteolytic activities in non-acid-producing *Aspergilli* [35,50,51]. The term “non-acid-producing *Aspergilli*” refers to *Aspergillus* spp. Including *A. oryzae* that generally produce proteolytic enzymes, and these become active at a neutral or alkaline PH value. Conclusively, the reduction in the production of a wide range of proteolytic enzymes supports the use of the *A. oryzae* BCC7051 *prtR*-disruption strain as a potential host for industrial protein production with low-proteolytic degradation.

Figure 2C presents the protease secretion performance of the deficient strain at different cultivation times, as indicated by the enzyme activity in the culture broth. In the parental strain, all protease activities were highest in the culture grown for 3 days, during the late logarithmic or early stationary cell growth phase, and tended to decrease after entering the stationary phase (5-day cultivation). In contrast, the extracellular protease activities of the deficient strain slowly increased with increasing culture time extension, yielding lower maximal enzyme activities than those of the control strain during the 5-day culture. The reduced secretion of protease in the culture broth coincided with the growth phenotype of the *AoprtR*-deficient strain grown on skim milk agar without a clear zone (Figure 1A, left panel). From the secretion results, the AoPrtR regulator could play a crucial role in extracellular protease production, which is active at different pH values based on pH specificity in the protease activity measurement assays.

**Figure 3:**
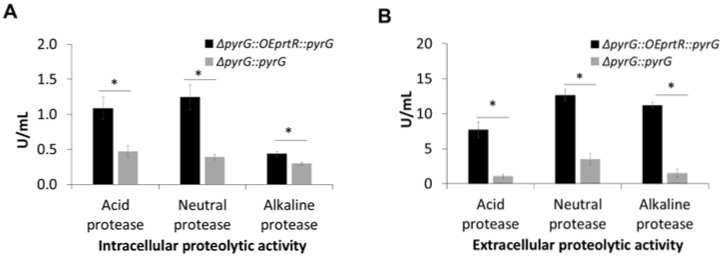
Proteolytic activities of *AoprtR*-overexpressing *A. oryzae*. (**A**) Intracellular and (**B**) extracellular proteolytic activities of *AoprtR*-overexpressed (Δ*pyrG::OEprtR::pyrG* as black bars) and parental (Δ*pyrG::pyrG* as gray bars) strains after 3 days of cultivation. Asterisks above the bars denote statistically significant differences in proteolytic activity between strains (*p* < 0.05). The data are represented as the mean value with standard error (mean ± SE).

To verify the role of AoPrtR in proteolytic enzyme production, we constructed an *AoprtR*-overexpressing strain (pOEAoPrtR overexpression plasmid construction, restriction enzyme analysis, and overexpression transformant screening are summarized in Figure S1C and Figure S1D, as well as Figure S3B, respectively). We observed that *AoprtR* overexpression significantly enhanced proteolytic enzyme production (Figure 3A,B) both in the intracellular compartment and extensively in the surrounding medium (i.e., by 3–7-fold). These results demonstrate the function of the AoPrtR regulator in controlling proteolytic enzyme production in *A. oryzae* BCC7051. However, the mechanism by which the regulator affects the entire enzyme production mechanism in *A. oryzae* remains unclear. As our results demonstrated, despite *AoprtR* disruption, protease activities were still detected in the culture broth. Therefore, this regulator may not control all protease production in *A. oryzae*, and, in turn, other cellular mechanisms may exist for cooperative protease production regulatory processes.

### 3.3. Functional Role of AoPrtR in the Transcriptional Regulation of Protease-Encoding Genes in A. oryzae

To assess the regulatory role of AoPrtR in protease-encoding gene expression, we compared the transcriptional levels of 26 protease-encoding genes between deficient and parental strains. Using SignalP 6.0, we annotated 18 *A. oryzae* candidate genes as putative extracellular protease-encoding genes with predicted signal peptide sequences. In addition, based on the absence of a signal peptide sequence, we identified eight putative intracellular protease-encoding genes (Table 1).

**Figure 4:**
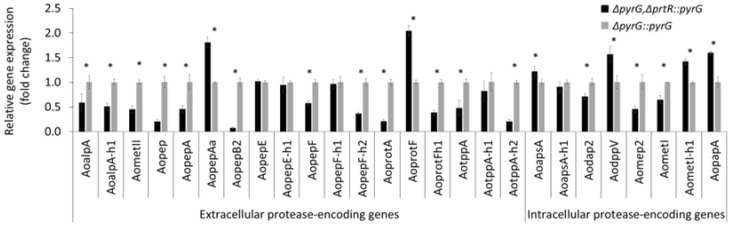
Effects of *AoprtR* disruption on the transcript levels of protease-encoding genes. RT-qPCR analysis of protease-encoding genes in AoprtR-deficient (Δ*pyrG*,Δ*prtR::pyrG* as black bar) and parental (Δ*pyrG::pyrG* as gray bar) strains. The relative gene expression was calculated as a fold change by normalizing the transcript level of the *AoprtR*-deficient strain to that of the parental strain, which was adjusted to one. Asterisks above the bars indicate statistically significant differences in the transcript levels of each gene among the strains (*p* < 0.05). The data are represented as the mean value with standard error (mean ± SE).

The RT-qPCR results revealed that *AoprtR* disruption positively and negatively affected protease transcription (Figure 4). AoprtR functional defects reduced the transcription of 15 protease-encoding genes. We observed significant transcriptional downregulation in the case of 12 extracellular protease-encoding genes (80% of the total of the candidate genes) in the deficient strain. Among these, we defined eight genes that encoded acid proteases: *Aopep*, *AopepA*, *AopepB2* (*pipA*), *AopepF*, *AopepF-h2* (*ocpH*), *AoprotA* (*sepI*), *AotppA* (*tppB*), and *AotppA*-*h2* (*tppA*). Remarkably, the transcriptional levels of *Aopep*, *AopepB2*, and *Aotpp-h2*, which encode extracellular serine protease, glutamic protease, and serine peptidase, respectively, decreased by >90%. *AopepB* and *AotppA* transcriptional downregulation was previously described in *A. niger* [35,49], *A. fumigatus* [41], *A. oryzae* RIB40 [26,36], and *P. oxalicum* [31]. However, no studies have reported the regulation of prtR-dependent *pep* in *Aspergilli.* In addition, the AoPrtR-mediated transcriptional regulation of the *AopepA* and *AopepF* acid-protease-encoding genes was in good agreement with the results of previous studies on *A. niger* [35,49]. *AoprtR* disruption also downregulated four extracellular non-acid-protease-encoding genes. These genes, including *AoalpA*, *AoalpA-h1*, *AometII* (*deuA*), and *AoprotF-h1*, were AoPrtR regulation targets, as indicated by the significantly reduced transcript levels in the deficient strain indicated (Figure 4, extracellular protease panel). Other studies have described significant downregulation of *alpA*, an alkaline-protease-encoding gene found in various *Aspergillus* species [30,33,34,41]. Therefore, the deletion of AoprtR reflected in the decrease in extracellular non-acid-protease-encoding gene transcription strongly supports the reduction in non-acid protease activities (Figure 2B).

Beyond AoPrtR-mediated extracellular protease-encoding gene regulation, three intracellular non-acid-protease-encoding genes’ (*Aodap2*, *Aomep2*, and *AometI1*) transcriptional levels were significantly reduced in the *AoprtR*-deficient strain (Figure 4, intracellular protease panel) in the absence of the AoPrtR protein. *dap2* encodes for an intracellular membrane-bound vacuolar protease involved in secretory pathway proteolysis; the deficient *prtR* may thus reduce the degradation of secreted proteins. The expression of these genes was downregulated as a result of AoprtR disruption, coinciding with reduced protease production and secretion (Figure 2A). In turn, *AoprtR* overexpression revealed the regulatory function that enabled the activation of protease-encoding genes, resulting in enhanced overall protease activity (Figure 3). These results indicate that AoPrtR is a putative protease activator that regulates a wide range of extracellular protease-encoding genes, particularly extracellular acid-type protease-encoding genes, followed by extracellular and intracellular non-acid-type protease-encoding genes in *A. oryzae* BCC7051. PrtT reportedly acts as an acid protease activator in certain *Aspergillus* spp. and *P. oxalicum* [30,31,34,41,52,53]. However, the function of PrtT as a non-acid protease activator in other fungi requires verification.

In contrast, *AoprtR* disruption enhanced the transcriptional levels of seven protease-encoding genes, four of which (i.e., *AoapsA*, *AodppV*, *AometI-h1*, and *AopapA*) encode intracellular non-acid proteases, displaying a 22–60% transcript level increase upon *AoprtR* disruption compared to the parental strain (Figure 4, right panel). As the intracellular neutral and alkaline protease activity reduction was below that of the extracellular activities, the transcriptional level increase could explain the correlation of the altered intracellular non-acid-protease-encoding gene regulation upon *AoprtR* disruption (Figure 2A,B). Therefore, the AoPrtR regulation defect led to increased expression of these genes, suggesting that AoPrtR functions as a putative repressor. The regulation of PrtT/PrtR by dipeptidyl aminopeptidase V-encoding *dppV* (*dppF*) differs among fungal species. Previous studies have described *dppV* repression in *P. oxalicum* and *A. niger* PrtT-deficient strains using transcriptomic analysis [31,53], whereas an increasing or unchanged transcription level was observed in *A. oryzae* RIB40, depending on the cultivation conditions [36]. Remarkably, *AoprtR* disruption enhanced the transcriptional levels of *AopepAa* (*pepA1*) and *AoprotF1* (*ocpB*), which encode for extracellular aspartic protease and serine carboxypeptidase, by 81% and 100%, respectively (Figure 4, left panel). However, the transcript levels of both genes were unchanged in the deficient *A. oryzae* RIB40 strain [36]. Notably, the high expression levels of both genes in the deficient strain might compromise the decrease in extracellular acid and alkaline protease activities, as shown in Figure 2C (left and right panel, respectively). In summary, PrtT/PrtR-mediated gene expression regulation may be *Aspergillus* species- and individual strain-dependent, including growth conditions. Finally, the expression of five protease-encoding genes (*AopepE*, *AopepE-h1*, *AopepF-h1*, *AotppA-h1*, and *AoapsA-h1*) remained unaltered, suggesting AoPrtR-independent control. This result suggests additional transcription factors that influence the expression of proteases in *A. oryzae* BCC7051. Alternative regulatory mechanisms involved in protease production in filamentous fungi, including *A. oryzae*, have been reported [30,42,54,55,56]. However, their relevance to the PrtR-dependent pathway in *A. oryzae* BCC7051 requires further exploration. Apart from the regulatory role of AoPrtR in protease-encoding gene expression, identifying a possible transcription regulation of the AoPrtR has become of interest. According to the literature reviewed by Numazawa et al. [36], the transcription of PrtR was promoted by the FlbC transcription factor, in addition to being influenced by nitrogen sources in the culture medium. This information could serve as a guide for the further discovery and study of a regulator of AoPrtR in *A. oryzae* BCC7051.

Taken together, our results reveal the role of AoPrtR in the transcriptional control of various acid-, neutral-, and alkaline-protease-encoding genes in *A. oryzae* BCC7051, coinciding with the protease activities observed in the *AoprtR*-deficient and overexpressed strains. We suggest that AoPrtR regulation in *A. oryzae* BCC7051, which is involved in the transcriptional activation or repression of a given gene set, might, under certain circumstances, be responsible for modulating proteolytic functions based on the pH conditions during measurement of protease activity. Such exploration is necessary for a thorough comprehension of fungal physiology during growth and development on proteinaceous substrates. Moreover, our results suggest that PrtR-dependent and perhaps other regulatory mechanisms also participate in the protease production of *A. oryzae*. Further insights into the regulatory mechanisms underlying protease production and secretion in *A. oryzae* could pave the way for fundamental research and industrial applications for precision bioprocessing of desired proteins.

### 3.4. Effect of Protease Deficiency on Enzyme Secretion by A. oryzae BCC7051

**Figure 5:**
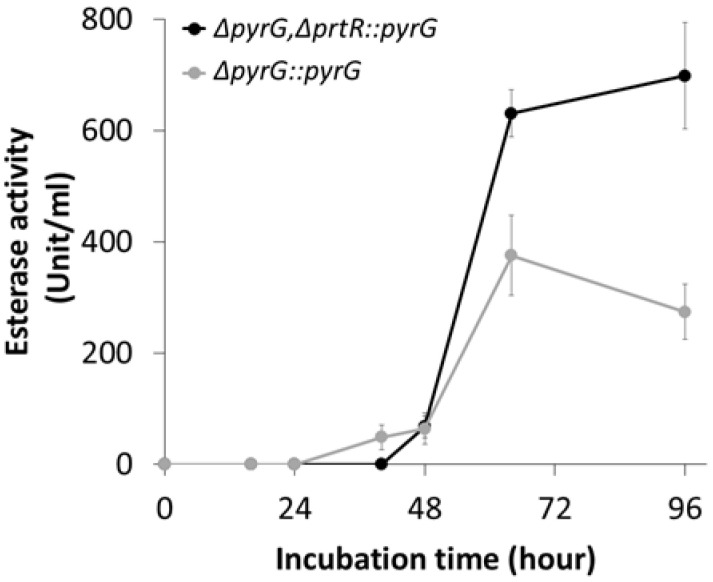
Extracellular esterase production by the AoprtR-deficient *A. oryzae* strain. The esterase secretion profiles of the AoprtR-deficient (Δ*pyrG*,Δ*prtR::pyrG* as black line) and parental (Δ*pyrG::pyrG* as gray line) strains grown for 96 h. The data are represented as the mean value with standard error (mean ± SE).

Abundant carboxylesterase-encoding genes allow *A. oryzae* to predictably secrete esterases as dominant enzymes, making it an attractive candidate for industrial production. Therefore, we hypothesized that extracellular protease deficiency caused by *AoprtR* gene disruption increases enzyme secretion. To assess the effect of protease deficiency on esterase secretion in *A. oryzae*, we performed a comparative time-course analysis of *AoprtR*-deficient and parental strain-derived extracellular esterases. Esterase secretion from all strains increased after 48 h of culture (Figure 5). The secretion rate in the disruption strain at 64 h was 630.87 ± 42.48 U/mL, which was 1.68-fold higher than that in the parental strain. Moreover, esterase secretion in the disruption strain remained remarkably stable through 96 h, whereas it decreased by 1.37-fold from 64 h. At the end of the culture, the disruption strain exhibited an esterase activity of 698.46 ± 95.59 U/mL, representing a 2.55-fold (155%) increase compared to that of the parental strain. These results confirm a reduction in protease activity, remarkably supporting the secretion of lipid-degrading enzymes such as esterases, reducing concerns about restricted harvest times, and allowing for the avoidance of fungal cell-derived protease activities during fermentation. Therefore, AoprtR disruption in the *A. oryzae* cell chassis demonstrates its potential to enhance enzyme secretion in industrial applications.

## 4. Conclusions

In this study, we identified the *AoprtR* ortholog in the genome of *A. oryzae* BCC7051, which is classified as a Zn(II)2-Cys6-type transcription factor family member. AoPrtR was shown to affect protease activities, as demonstrated by *AoprtR* disruption and overexpression experiments. We explored the functional role of AoPrtR in the transcriptional regulation of acid-, neutral-, and alkaline-protease-encoding genes through gene disruption, the results of which were in good agreement with the enzyme activities in mycelial and cell-free broth cultures. AoPrtR was a transcription activator that significantly regulated extracellular acid-protease-encoding genes. Notably, it also repressed intracellular non-acid-protease-encoding genes. In addition, we identified certain protease-encoding genes that exhibited unaltered transcriptional changes in the *AoprtR* disruption strain, thereby suggesting the existence of an indirect mechanism for controlling gene expression relevant to PrtR function. These results could be applied to improve the *A. oryzae* strain, providing a potential cell platform for protein production and secretion supported by extracellular esterase production via protease-encoding gene expression regulatory mechanisms. The findings of this study offer an opportunity to enhance protein production capabilities with cost-effective and sustainable production bioprocesses to meet the demands of biotechnological industries based on synthetic biology and precision fermentation.

## Figures and Tables

**Table 1:** Selected putative protease-encoding genes of *A. oryzae* BCC7051.

Gene Name	Gene ID	Protease Classification	Protease Type	Signal Peptide Sequence
*AoalpA*	XP_001820144.1	Serine protease	Alkaline	Yes
*AoalpA-h1*	XM_001824768	Serine protease		Yes
*AometII*	OOO07665.1	Metalloprotease	Neutral	Yes
*Aopep*	OOO13707.1	Serine protease	Acid	Yes
*AopepA*	XP_001824175.1	Aspartic protease	Acid	Yes
*AopepAa*	XP_001727263.1	Aspartic protease	Acid	Yes
*AopepB2*	XM_001820894	Glutamic protease	Acid	Yes
*AopepE*	XP_001819842.1	Aspartic protease	Acid	Yes
*AopepE-h1*	XM_023235801	Aspartic protease		Yes
*AopepF*	XP_001824682.3	Serine carboxypeptidase	Acid	Yes
*AopepF-h1*	XM_001823919	Serine carboxypeptidase		Yes
*AopepF-h2*	XM_001827461	Serine carboxypeptidase		Yes
*AoprotA*	OOO12107.1	Serine protease	Acid	Yes
*AoprotF*	OOO05958.1	Serine carboxypeptidase	Neutral/weak alkaline	Yes
*AoprotF-h1*	XP_001820936.3	Serine carboxypeptidase		Yes
*AotppA*	XP_001825903.1	Serine peptidase	Acid	Yes
*AotppA-h1*	XM_001818255	Serine peptidase		Yes
*AotppA-h2*	XM_001822814	Serine peptidase		Yes
*AoapsA*	XP_001821168.1	Metalloprotease	Neutral	No
*AoapsA-h1*	XM_001822732	Metalloprotease		No
*Aodap2*	XP_001824982.1	Serine protease	Neutral/weak alkaline	No
*AodppV*	OOO13054.1	Serine protease	Neutral/weak alkaline	No
*Aomep2*	XM_001825611	Metalloprotease	Neutral	No
*AometI*	XP_023089421.1	Methionine aminopeptidase	Neutral	No
*AometI-h2*	XM_023237785	Methionine aminopeptidase		No
*AopapA*	BAH84978.1	Serine protease	Neutral	No

*p*-value < 0.05. Signal peptide prediction using the SignalP 6.0 program.

## Data Availability

The original contributions presented in the study are included in the article/Supplementary Material, further inquiries can be directed to the corresponding authors.

## Supplementary Materials

The following supporting information can be downloaded at https://www.mdpi.com/article/10.3390/jof11010006/s1. Table S1: Overlapping primers used for plasmid construction by yeast assembly; Table S2: Oligonucleotide primers used for the screening of gene disruption; Table S3: Oligonucleotide primers used for gene expression analysis; Table S4: pH values of the culture broth during 5 days of incubation; Figure S1: Construction of pDAoprtR disruption and pOEAoprtR overexpression plasmids, and restriction enzyme analysis; Figure S2: Phylogenetic tree from the multiple sequence alignment of the prtT/prtR protein of *Aspergillus* spp., *Penicillium* spp., and entomopathogenic fungi generated by CLUSTAL Omega (1.2.4); Figure S3: *AoprtR* gene disruption and overexpression in *Aspergillus oryzae*; Figure S4: RT-PCR analysis of *AoprtR* gene.
