# Supplementary material for: Development of Aspergillus oryzae BCC7051 as a Robust Cell Factory Towards the Transcriptional Regulation of Protease-Encoding Genes for Industrial Applications
Source: J Fungi (Basel). 2024 Dec 25;11(1):6. doi: 10.3390/jof11010006 (PMC11765966; doi:10.3390/jof11010006)
Supplement: Supplementary file 1 [file jof-11-00006-s001.zip › jof-3317199-supplementary.pdf]

Supplementary data

Development of *Aspergillus oryzae* BCC7051 as a Robust Cell Factory Towards the Transcriptional Regulation of Protease-Encoding Genes for Industrial Applications

Sarocha Panchanawaporn, Chanikul Chutrakul *, Sukanya Jeennor, Jutamas Anantayanon and Kobkul Laoteng

Functional Ingredients and Food Innovation Research Group (IFIG), National Center for Genetic Engineering and Biotechnology (BIOTEC), National Science and Technology Development Agency (NSTDA), Thailand Science Park, Phahonyothin Road, Khlong Nueng, Khlong Luang, Pathum Thani 12120, Thailand

Correspondence: chanikul@biotec.or.th; Tel.: +66-25646700 (ext. 3769)

Table S1 Overlapping primers used for plasmid construction by yeast assembly

Plasmid	Amplified fragment	Sense primer name	Sequence (5' to 3')	Antisense primer name	Sequence (5' to 3')
pDAoprtR	5' flanking (1.4 kb)	F1	CGCGTTGGCCGATTCATT CCCGGGCGCGCCGGTTG GAATATCCGAGAATC	R1	GGTACCTAGCTAGTTAGCA AGAATTCACAAGCGGTCATT GAACGTCTAATGC
	3' flanking (1.3 kb)	F2	AAGTACCTACGTACGGAC TTAAGCTTCAGCGCAGTC GACGGTTCTC	R2	CGTTGTAAAACGACGGGAT CGCGGCGCGCCATCTGGCA TTGGACAGTGAG

Table S2 Oligonucleotide primers used for the screening of gene disruption

Genotypic analysis	Size (kb)	Sense primer name	Sequence (5' to 3')	Antisense primer name	Sequence (5' to3')
Δ*AoprtR::AopyrG*	Parental = 0.0<br>Disruptant = 2.5<br>(5' UTR)	F3	GCTGAGGCATACCC GAAGCCAAGCTC	R3	CACCTCTTGGCAAAT CGAACAGCGGACTG
	Parental = 0.0<br>Disrupant = 2.2<br>(3' UTR)	F4	TCCCGTGGAGGCGG CGAAGCAGTACCAG	R4	GCTACCTAGGGTATT GTATCCGACTGAG

Table S3 Oligonucleotide primers used for gene expression analysis

Gene name	Sense primer name	Sequence (5' to 3')	Antisense primer name	Sequence (5' to 3')
AoalpA	AoalpA_F	CTACAAGATCAACAAGTTCGC	AoalpA_R	ATGTAGTCGGTGCTCTGCTG
AoalpA-2	AoalpA-2_F	GGTCCCTAGCGGGCTACTC	AoalpA-2_R	CCGAACGAGAGGCTGTCAC
Aomep-2	Aomep-2_F	GGATAATGGTGTGGCACACG	Aomep-2_R	TGGGTGATGCTGGGAACAG
AometII	AometII_F	GCTCTTCTCCTGGTCTTGAG	AometII_R	GTGGAGGCGATATCGAACTC
AopepA	AopepA_F	ACGGTACTGTCGTGACTTCTCC	AopepA_R	TGGAGGCGTTTCCAGAAGGCTTG
AopepB-2	AopepB-2_F	TCGTACTCCTCCACCAGTGG	AopepB-2_R	CAGTCTGACCACCAGCAGC
AopepF	AopepF_F	ATGCAGGTCTCTTGCCCAACTC	_AopepF_R	CACGAGTATGGGTTTCGAGCAG
AopepF-2	AopepF-2__F	GTGCTAGCTCCCTCGAAGG	AopepF-2_R	TCCCTGGACGTCTCCTCTTC
AopepF-3	AopepF-3_F	GGATGTAGCTCTCTCGAGG	AopepF-3_R	CACAAAGTCCTCGGCAGTC
AoprotA	AoprotA_F	CACAATGACTCGCGCTATGC	AoprotA_R	TCCAGAATCACTCCAATGCCG

AoprotF	AoprotF_F	CTGGTTGGTGCCGTCGCC	AoprotF_R	CCTGGCCCACCATTCATCC
AoprotF-2	AoprotF-2_F	GCACAGAGATGCTAGCTGAG	AoprotF-2_R	GGCTCGATTATCGGACCCG
AoprotF-3	AoprotF-3_F	CGATACGCTGGGGATTGTG	AoprotF-3_R	GCTGCTTGGCAGGCTATCG
AotppA	AotppA_F	TCGTTCCTGATCTGACCCAG	AotppA_R	CTGAGCGATCAGGTTGCAG
AotppA-2	AotppA-2_F	GACGTTGCTGCTCAGGCG	AotppA-2_R	GCCACGGGTTGAGAAATCC
AotppA-3	AotppA-3_F	TGGACCACTGCTTCCCTTG	AotppA-3_R	GAGTTAGGCTTCGACAGGAG
AopepE	AopepE_F	ATGTGCAGGCTCTCGGCCAG	AopepE_R	TAGGTTTGAGCTGCCAGTGTC
AopepE-3	AopepE-3_F	TCACCCAATACGGCCGCG	AopepE-3_R	CCGGTTCCATAACCCACGC
Aopep	Aopep_F	TCCTCGGTGGTTGACGGA	Aopep_R	TCCTGCGCGTATAGACCAG
AoapsA	AoapsA_F	CACCAATGTCAAGCCGGTC	AoapsA_R	TCGGCGAACTTGACGGTAG
AoapsA-2	AoapsA-2_F	TGAGAGAGACGGCAGTAGC	AoapsA-2_R	GAGGCCCTTCGTCGTGTAG
Aodap2	Aodap2_F	TGCGAGCGACAGGACAGG	Aodap2_R	GGAGATCTAGCTGTCGAGC
AodppV	AodppV_F	CATAGTTGGGCATGCTCGAG	AodppV_R	CGATGTACGAGTCCCAGTG
Aom18	Aom18_F	CGCGAGACGTGGAACTCC	Aom18_R	CGCTCTGAGTGACACATGG
Aom18-2	Aom18-2_F	CGTGACTTGGGTGTCGCAG	Aom18-2_R	TCAGCAGTGCGGTTCAGCTC
AometI	AometI_F	ACAAGGAGGAGCAGGAAGG	AometI_R	CCGTCTTCAAGTGGGCGC
AometI-2	AometI-2_F	TAGCTCGGGAGGTACTGG	AometI-2_R	CACCATCGAGAAGAATCCGC
AopapA	AopapA_F	CCTACAGGGTGGACCTGG	AopapA_R	CAACGGCGAACGGCTTCAC
AopepAa	AopepAa_F	GTGCTGTTAGTGCTTCCTCG	AopepAa_R	ACGCATACGAGGCATCACC
AopepP	AopepP_F	ACGGCGTTCCAGACACGAC	AopepP_R	GCGCTGCGGAGGTTACATAT

Table S4 pH values of the culture broth during 5 days of incubation

Incubation time (day)	pH value	
	Parental strain (Δ*pyrG::pyrG*)	*AoprtR*-deficient strain (Δ*pyrG*,Δ*prtR::pyrG*)
0	5.58±0.01	5.56±0.01
1	3.88±0.06	3.94±0.01
2	5.37±0.04	4.97±0.64
3	5.97±0.01	5.93±0.00
4	5.73±0.04	5.87±0.04
5	5.76±0.04	5.80±0.04

A)

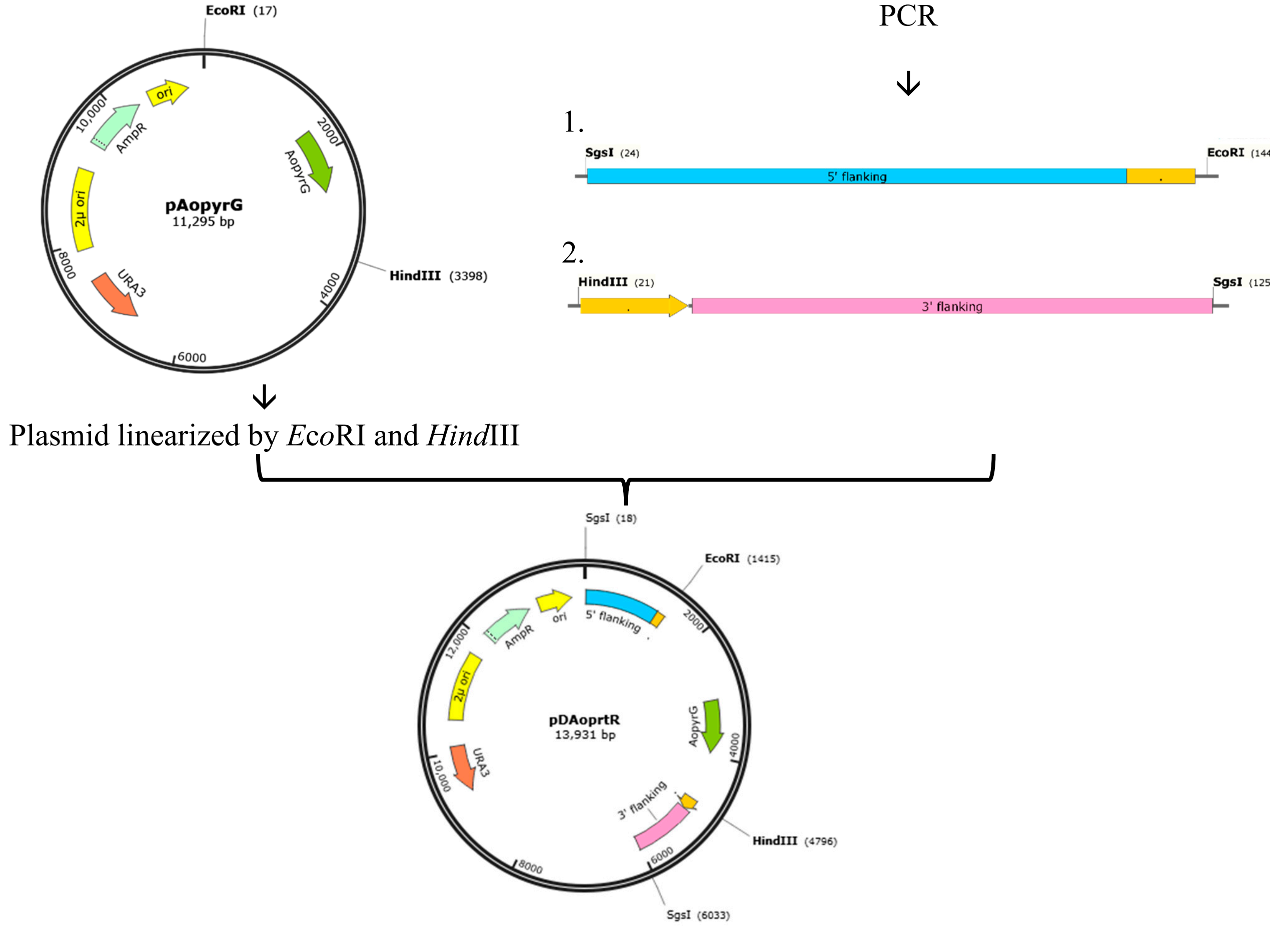
EcoRI (17)
ori
AmpR
10,000
2000
AopyrG
2μ ori
pAopyrG
11,295 bp
8000
URA3
4000
HindIII (3398)
6000
Plasmid linearized by *Eco*RI and *Hind*III
PCR
1.
SgsI (24)
5' flanking
EcoRI (144
2.
HindIII (21)
3' flanking
SgsI (125
SgsI (18)
EcoRI (1415)
ori
AmpR
5' flanking
2000
12,000
2μ ori
pDAoprtR
13,931 bp
AopyrG
4000
URA3
10,000
3' flanking
HindIII (4796)
8000
6000
SgsI (6033)

B)

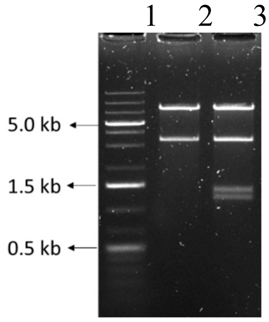
1
2
3
5.0 kb
1.5 kb
0.5 kb

C)

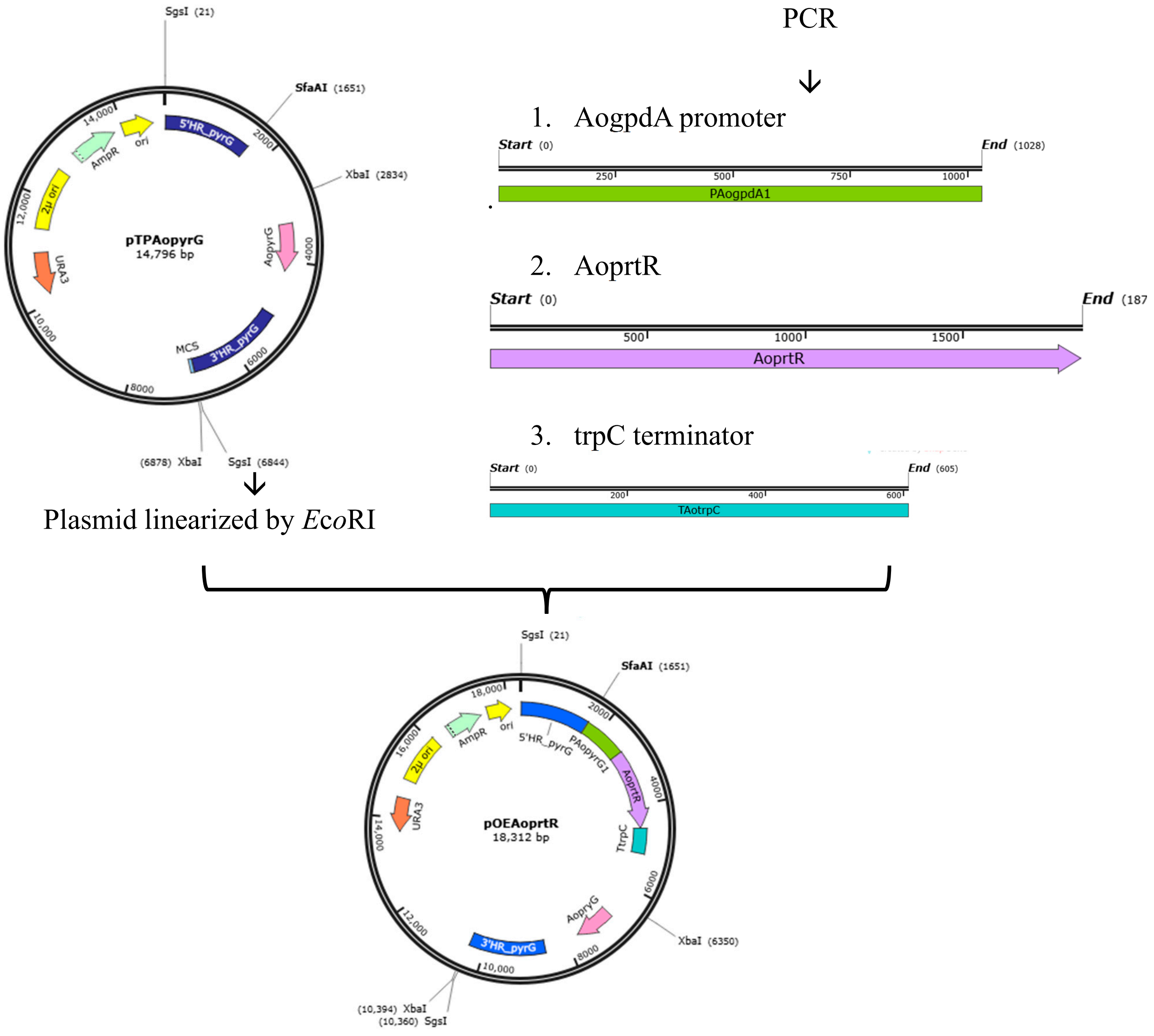

D)

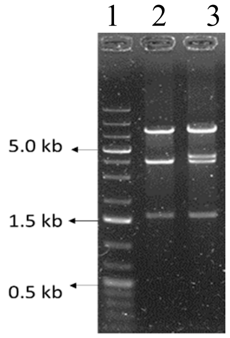

Figure S1 Construction of pDAoprtR disruption and pOEAoprtR overexpression plasmids, and restriction enzyme analysis. A) Schematic construction of the disruption plasmid. B)

Restriction enzyme analysis of the constructed disruption plasmid by gel electrophoresis. Lane 1 is a 1-kb DNA ladder. Backbone plasmid (lane 2: 3.4- and 8-kb) and the disruption plasmid (lane 3: 1.3-, 1.4-, 3.4- and 8-kb) were analyzed by *Eco*RI, *Hind*III and *Sgs*I digestion. C) Schematic construction of the pOEAoprtR. D) Restriction enzyme analysis of the constructed overexpressed plasmid by gel electrophoresis. Lane 1 is a 1-kb DNA ladder. Backbone plasmid (lane 2: 1.1-, 1.6-, 4.0- and 8.0-kb) and the overexpressed plasmid (lane 3: 1.1-, 1.6-, 4.0-, 4.6- and 8.0-kb) were analyzed by *SfaA*I, *Sgs*I and *Xba*I digestion.

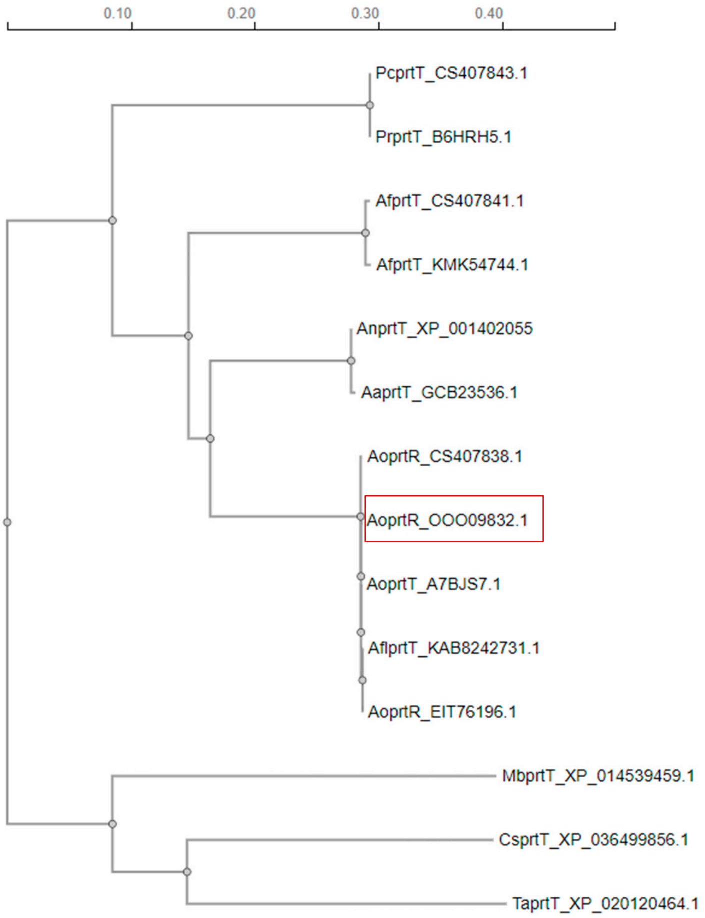

Figure S2 Phylogenetic tree from the multiple sequence alignment of the PrtR/PrtR protein of *Apergillus* spp., *Penicillum* spp. and entomopathogenic fungi generated by CLUSTAL Omega (1.2.4). The list of fungal PrtR/PrtR protein with GenBank accession numbers are described: PcprtT_CS407843.1 [*Penicillium chrysogenum*], PrprtT_B6HRH5.1 [*Penicillium rubens* Wisconsin 54-1255], AfprtT_CS407841.1 [*Aspergillus fumigatus*], AfprtT_KMK54744.1 [*Aspergillus fumigatus* Z5], AnprtT_XP_001402055 [*Aspergillus niger* CBS513.88], AaprtT_GCB23536.1 [*Aspergillus awamori* IFM58123], AoprtR_CS407838.1 [*Aspergillus oryzae* IF04177], AoprtR_OOO09832.1 [*Aspergillus oryzae* BCC7051 shown as red box], AoprtT_A7BJS7.1 [*Aspergillus oryzae* RIB40], AflprtT_KAB8242731.1 [*Aspergillus flavus* CBS121.62] and AoprtR_EIT76196.1 [*Aspergillus oryzae* 3.042]. The phylogeny is rooted with MbprtT_XP_014539459.1 [*Metarhizium brunneum* 4556], CsprtT_XP_036499856.1 [*Colletotrichum siamense* Cg363] and TaprtT_XP_020120464.1 [*Talaromyces atroroseus* IBT 11181]. The relative phylogenetic distance is measured by the scale on the top. The AoprtR_OOO09832.1 is boxed in red.

A)

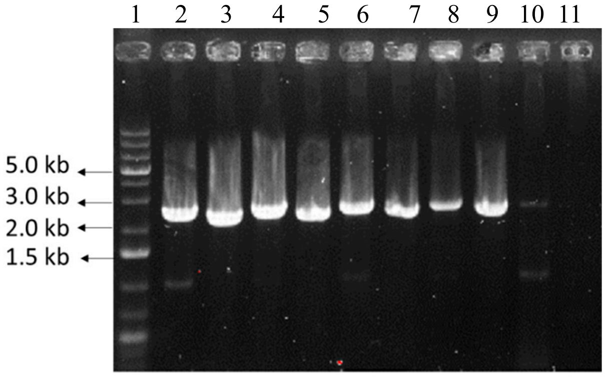

B)

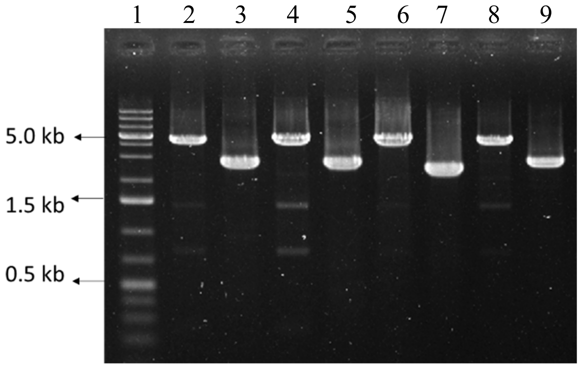

Figure S3 ***AoprtR*** **gene disruption and overexpression in** ***Aspergillus oryzae.*** A) Screening of disrupted transformants by PCR analysis. Lane 1 is 1-kb DNA ladder. A band of 2.5-kb fragment amplified from 5'UTR of disruptants no. 1, 2, 3, 4 are shown in lanes 2, 4, 6 and 8. A band of 2.2-kb fragment amplified from 3'UTR of disruptants is shown in lanes 3, 5, 7 and 9. No specific amplicon was obtained from the parental strain (lanes 10 and 11). B) Screening of overexpressed transformants. Lane 1 is 1-kb DNA ladder. The bands of 4.6- and 2.9- kb of 5'UTR (lanes 2, 4, 6, and 8) and 3'UTR (lanes 3, 5, 7, and 9) of overexpressed strains were amplified.

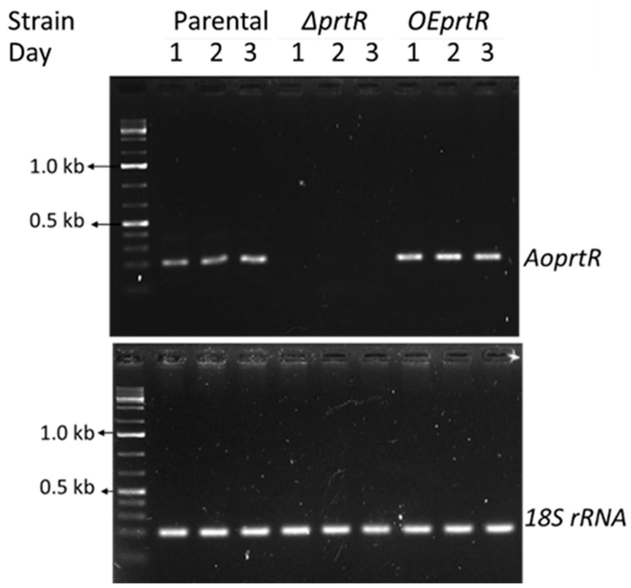

Figure S4 RT-PCR analysis of *AoprtR* gene. Investigation of *AoprtR* gene expression with a band of 0.2-kb fragment in the parental, *ΔAoprtR* and *OEprtR* strains by RT-PCR analysis at 24, 48, and 72 h (top panel). The *18S rRNA* was used as a housekeeping gene with a band of 0.2-kb fragment (bottom panel). The left lane is 100-bp DNA ladder.
